# *Candida albicans*: A Model Organism for Studying Fungal Pathogens

**DOI:** 10.5402/2012/538694

**Published:** 2012-09-29

**Authors:** M. Anaul Kabir, Mohammad Asif Hussain, Zulfiqar Ahmad

**Affiliations:** ^1^Molecular Genetics Laboratory, School of Biotechnology, National Institute of Technology Calicut, Calicut 673601, Kerala, India; ^2^Biomedical Engineering Option, Department of Electrical and Computer Engineering, Faculty of Engineering, King Abdulaziz University, P.O. Box 80204, Jeddah 21589, Saudi Arabia; ^3^Department of Biological & Environmental Sciences, Alabama A & M University, Normal, AL 35762, USA

## Abstract

*Candida albicans* is an opportunistic human fungal pathogen that causes candidiasis. As healthcare has been improved worldwide, the number of immunocompromised patients has been increased to a greater extent and they are highly susceptible to various pathogenic microbes and *C. albicans* has been prominent among the fungal pathogens. The complete genome sequence of this pathogen is now available and has been extremely useful for the identification of repertoire of genes present in this pathogen. The major challenge is now to assign the functions to these genes of which 13% are specific to *C. albicans*. Due to its close relationship with yeast *Saccharomyces cerevisiae*, an edge over other fungal pathogens because most of the technologies can be directly transferred to *C. albicans* from *S. cerevisiae* and it is amenable to mutation, gene disruption, and transformation. The last two decades have witnessed enormous amount of research activities on this pathogen that leads to the understanding of host-parasite interaction, infections, and disease propagation. Clearly, *C. albicans* has emerged as a model organism for studying fungal pathogens along with other two fungi *Aspergillus fumigatus* and *Cryptococcus neoformans*. Understanding its complete life style of *C. albicans* will undoubtedly be useful for developing potential antifungal drugs and tackling *Candida* infections. This will also shed light on the functioning of other fungal pathogens.

## 1. Introduction

*Candida albicans* is an opportunistic fungal pathogen that exists as a harmless commensal in the gastrointestinal and genitourinary tracts in about 70% of humans and about 75% of women suffer from *Candida* infection at least once in their lifetime [1–4]. However, it becomes opportunistic pathogen for immunocompromised patients, for some immunologically weak individuals, or even for healthy persons. The infection caused by *C. albicans* is commonly known as candidiasis. Candidiasis can be classified into two categories depending upon the severity of the disease. In the first category are the mucosal infections and the best known among these mucosal infections is thrush which is characterized by white spots in the infected membranes. These infections generally affect gastrointestinal epithelial cells, vaginal, or oropharyngeal mucosa. Furthermore, Vulvo Vaginal Candidiasis (VVC) is quite common among women, and some of them experiences repeated occurrences of this infection, which is known as Recurrent Vulvo Vaginal Candidiasis (RVVC). However, it causes life-threatening, systemic infections to severely ill patients in whom mortality rate is about 30% [5–8]. Systemic *Candida* infections are common to immunocompromised individuals, including HIV-infected patients, transplant recipients, chemotherapy patients, and low-birth weight infants [9, 10]. Although some non-*albicans* species like *Candida glabrata, Candida krusei, Candida dubliniensis, Candida parapsilosis,* and *Candida tropicalis *are recovered from infected individuals, *C. albicans* remains a major infectious fungal agent [11]. Historically, *C. albicans* is known to us since 400 BC when the renowned Greek physician, Hippocrates, identified a microbial infection and he named it as “thrush,” which is caused by this pathogen [12]. However, it was not studied like any other model organisms till late twentieth century and early studies were mainly confined to the identification of *C. albicans* strains [13–15].

With improvements in healthcare system worldwide, the number of elderly people and immunocompromised patients has been increased dramatically and so the infections caused by various microbes. It has been observed that *Candida* species are one of the four most common causes of bloodstream and cardiovascular infections in US hospitals [7, 16]. Bloodstream infections caused by *Candida* are responsible for as high as 50% mortality rate among the infected patients [16–18]. In case of neonatal care units, *Candida* related bloodstream infections are even more frequent [19]. Because of the above-mentioned reasons, *C. albicans* has gained importance as a potential human pathogen, which warranted detailed study of this organism to understand its biology.

In the 1970s and 1980s, some *Saccharomyces cerevisiae* laboratories started working on *C. albicans,* and in the 1990s, a large number of yeast laboratories switched to study different aspects of *C. albicans* resulting in the initiation of genome sequencing of this pathogen in 1996. The completion and availability of genome sequence of *C. albicans* in 2004 made possible to initiate rigorous research activities and expanded our knowledge of this important pathogen [20]. In the last two decades, substantial advances have been made in understanding pathogenicity, genome structure and dynamics, pattern of gene expressions in different conditions, drug resistance, biofilm formation, and host-parasite interactions. Undoubtedly, it has emerged as a member of elite group of model organisms at least for fungal pathogens. Here we would like to discuss briefly some of the important features of *C. albicans,* which are being studied vigorously to understand its complete biology and how it has elevated to the level of model fungal pathogen. 

## 2. Techniques for Studying *Candida albicans*

Transformation of intact cell by exogenous DNA has been a major challenge for different cell types starting from bacteria to human cell line. In case of *C. albicans*, most of the techniques used for genomic and proteomic analyses are directly transferred from the model organism, the budding yeast *S. cerevisiae*. In the beginning, *Candida* research suffered a great deal due to its diploid nature in which genetic manipulation was not amenable. However, in the last two decades, several important techniques have been developed and applied to the genetic manipulation and proteomic studies with respect to its interaction with host, biofilm formation, drug resistance, morphogenetic states, phenotypic switching, and many other aspects. Current techniques, which are used in molecular genetic studies include DNA transformation, sequential rapid gene disruption, RNA isolation, RNA-sequencing, epitope tagging, use of reporter genes and regulatory promoters, chromatin immunoprecipitation, and DNA microarray. Here we briefly discuss two important techniques, that have been developed to study other organisms and they are being used for *Candida* biology as well. These technologies have enormous potential to uncover a myriad of interesting and astonishing biological phenomena. 

### 2.1. RNA-Sequencing

RNA sequencing (RNA-seq) is a recently developed technology, which has been applied to study the transcriptomes of different organisms starting from bacteria to human. Understanding the transcriptome is a prerequisite to get full view of functional elements of the genome, which will lead to interpret and understand the molecular details of cellular constituents, developmental processes, and diseases. In brief, total RNAs isolated from different cells are converted into a library of cDNA fragments and adapters are attached to one or both the ends of these cDNA molecules. Subsequently, each cDNA molecule is sequenced by high-throughput sequencing technology to obtain short sequences of sizes ranging from 30 to 400 bp depending upon the sequencing technology used [21]. In principle, any high-throughput sequencing technology can serve this purpose. The high-throughput technology such as Illumina IG, Applied Biosystems SOLiD, Roche 454 Life Science, and Helicos Biosciences tSMS systems are being used for a wide variety of transcriptome analysis [22–28]. For example, these technologies have been applied to a range of model organisms such as *S. cerevisiae, S. pombe, A. thaliana, D. melanogaster*, mouse, and human for detailed analysis of transcribed regions of the corresponding genomes [29–34]. This new and innovative technology is also used for *C. albicans* transcriptome analysis. Recently, Bruno et al. have adopted RNA-sequencing method to generate a high-resolution map of transcriptome of this pathogen under several different environmental conditions [35]. Interestingly, this study revealed 602 novel transcriptionally active regions (TARs) in the *Candida* genome and numerous novel introns as well [35]. In another study, Mitrovich et al. have analyzed noncoding small nucleolar RNA (snoRNA) genes and revealed an alternative mechanism for widespread intron loss [36]. Application of this method in several other conditions with respect to adherence, disease propagation, biofilm formation, yeast-hyphae transition, and drug resistance will certainly provide an insight into the functioning of this organism in great detail. 

### 2.2. Chromatin Immunoprecipitation (ChIP)

The advent of chromatin immunoprecipitation (ChIP) has contributed greatly to the understanding of molecular mechanisms of different pathways in which DNA-protein interactions are involved. Chromatin immunoprecipitation assay was developed in the middle of 1980s to monitor the association of RNA polymerase with the transcribed regions of genomes in *Escherichia coli *and *Drosophila melanogaster* [37–39]. In early ChIP assays, UV irradiation was used for cross-linking DNA and proteins associated with it, and later Solomon et al. pioneered the use of formaldehyde as a cross-linking agent [40]. Soon thereafter, ChIP has been extensively used for studying the localization of posttranslationally modified histone proteins and their variants, chromosome-associated proteins, chromatin modifying enzymes, and also for the identification of target DNA sequences for large number of transcription factors and repressors [41]. Moreover, the combination of ChIP with DNA microarray and high-throughput DNA/RNA sequencing technologies has revolutionized functional genomics as never before. This has enabled the characterization of many transcription factors and other proteins on a genomewide scale for a variety of organisms [42–44]. A brief description of the principle of ChIP is given here. Generally, DNA and proteins are reversibly crossed-linked with formaldehyde so that proteins will covalently attach to DNA target sequences. Subsequently, the chromatins are fragmented either by micrococcal nuclease (i.e., MNase) or by sonication of the whole cells or isolated nuclei so that the DNA fragments of sizes 200–1000 bp can be generated with average value of 500 bp. Then the chromatins are immunoprecipitated with specific antibody that is generated against a protein of interest. Immunoprecipitated complexes are washed to remove nonspecific proteins, DNA-protein cross-link is reversed and ChIP-enriched DNA is purified. Purified DNA can be subjected to direct high-throughput sequencing (ChIP-seq) to determine the exact sequence of the DNA present in the immunoprecipitated chromatin [45]. DNA can also be cloned into plasmid vectors and then sequencing can be performed [46, 47]. Moreover, DNA sequences can also be identified by labeling and hybridization to genome-wide or tiling microarrays, end-point polymerase chain reaction (PCR), or with quantitative PCR [48–50].

This technology has been applied to *Candida* biology as well in many laboratories, and many novel findings are obtained. For example, Sellam et al. have used this ChIP-chip method to characterize Ndt80p fluconazole-dependent regulon and identifying its key role as activator for ergosterol metabolism genes [51]. Recently, Lassak et al. have used *In Vivo* genome wide ChIP-chip and *In Vitro* footprint analyses to reveal the Efg1 sequences (EGR-box, TATGCATA) in both yeast and hyphal forms for *C. albicans* [52]. This method has also been applied to understand different regulons, such as Cap1p regulon and Tac1p regulon for this pathogen [53, 54]. Furthermore, ChIP and its different variants are used for the study of *C. albicans* along with other important fungal pathogens. This clearly suggests that *C. albicans* is now being considered as an important pathogen that needs thorough investigation into its functioning at molecular level.

## 3. Genetics of *Candida albicans*

The early studies on the genetics of *C. albicans* involved mainly the isolation of auxotrophic mutants and UV was the major source for mutagenesis [55]. The rationale behind those studies was to determine whether there is any correlation between the change in phenotypes and change in virulence. During these studies, it was observed that this organism is diploid nature. The diploid characteristic of this pathogen was deduced in early 1980s by the findings that many clinical isolates showed strongly biased auxotrophic characters after UV irradiation [56, 57]. This observation led to the hypothesis that natural heterozygosity exists for some loci of *C. albicans*. This was later substantiated by the analyses of sectored colonies obtained from UV irradiation of putative natural heterozygotes and also from revertants of mutants generated from chemical mutagenesis [58–60]. In addition, determination of DNA content and optical assays for DNA reassociation kinetics proved that *C. albicans* is indeed a diploid organism [61, 62]. Due to this diploid nature of this pathogen, it was not easily amenable to genetic manipulation and research on this pathogen suffered a great deal in the beginning. Furthermore, genetic analysis of this pathogen suffered because of its heterozygous nature and chromosomal instability. However, it has been postulated that high level of heterozygosity in *C. albicans* might play an important role in achieving diversity within the species which might be required for its survival in different adverse conditions. This aspect has been extensively reviewed by Larriba and Calderone [63]. Genetic mapping of this pathogen began in the 1980s by applying spheroplast fusion method, and subsequently, recombination analyses were performed [64, 65]. This approach was adopted due to the fact that the conventional forward genetic methods could not be possible because of its asexual nature. 

It should be noted that generally yeast genetics has been studied with two sexually, well-characterized yeasts such as *S. cerevisiae* and *Schizosaccharomyces pombe* rather than asexual *C. albicans* [66]. Now the question is why this pathogen is considered to be asexual. Perhaps the lack of identification of mating forms in the laboratory strains led to this conclusion. However, this idea has been challenged by the identification of the *M*ating *T*ype-*L*ike Locus or *MTL* which is very much similar to *MAT* locus of *S. cerevisiae* [67]. For *S. cerevisiae*, *MAT* locus exists as *MATa* or *MAT*α** alleles that determine the mating type of haploid cells through the expression of a set of transcriptional factors [68, 69]. Two haploid cells harboring *MATa* and *MAT*α** alleles will mate with each other (cells carrying the same mating locus will not mate) and produce diploid that cannot mate further. Instead, under appropriate nutritional conditions, the diploid cells will sporulate and produce haploid cells of a and *α* mating types. On the other hand, due to its diploid nature, *C. albicans* strains do not mate. Also, it has not been proved so far that *C. albicans* undergoes meiosis and no haploid version of this strain is available. However, after the identification of *MTL*, two laboratories constructed *C. albicans* strains carrying only one allele of *MTL* [70, 71]. This was achieved by disrupting opposite allele or by selecting the strains for loss of one copy of chromosome 5 in which *MTL* resides. The resulting a and *α* strains were able to mate and produced tetraploid both in the laboratory media and in mouse model; however, the frequency was quite low. The tetraploid cells can undergo concerted loss of chromosomes and significant portion of cells can revert back to diploid state and thus, complete the parasexual cycle [70–73]. On the other hand, *S. cerevisiae* can perform meiosis and complete sexual cycle; however, similar mechanism was not identified in *C. albicans*. 

Another important feature required for mating in *C. albicans* is epigenetic switch of white-opaque transition, which was first reported by Slutsky et al. [74]. It has been demonstrated that cells which are homozygous or hemizygous in *MTL* can undergo white-opaque switch and only opaque cells are able to mate at higher frequency [75]. Furthermore, chromosomal instability is an important attribute of *C. albicans* and perhaps plays a significant role for generating different phenotypes including white-opaque switching required for its mating. The plasticity and dynamic nature of *Candida* genome has been well documented by several researchers [76–79].

## 4. Is Yeast-Hyphae Transition Crucial for Virulence? 

**Figure 1:**
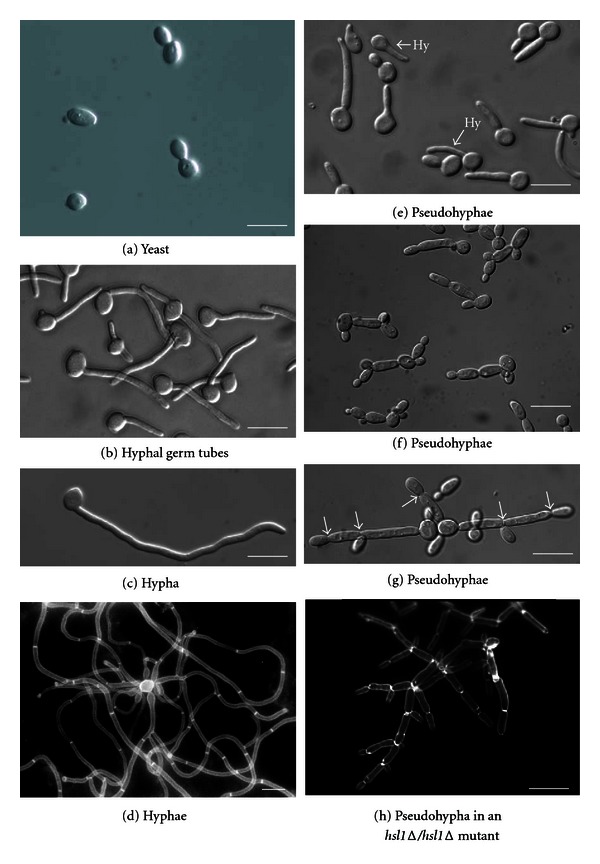
Yeast, hyphal and pseudohyphal morphologies. (a) Budding yeast cells appear similar to diploid *Saccharomyces cerevisiae* cells. Shortly after inoculation of unbudded yeast cells, (b) hyphal germ tubes are narrower and more uniform than (e) pseudohyphal buds, which have a constriction at the bud neck. However, it is difficult to obtain a population that consists solely of pseudohyphal cells; in the conditions used, 25% of the cells are hyphal, examples of these are indicated by an arrow plus “Hy.” (c) After 180 minutes, hyphae continue to display parallel-sided walls with no constrictions or branches. (d) Mature hyphal mycelia are shown. (e) Pseudohyphae exhibit morphologies ranging from short pseudohyphae that appear to be polarized yeast cells to (g) two long pseudohyphae that superficially resemble hyphae. (h) The mature pseudohyphal mycelium that results from a homozygous hsl1D mutation is shown. All forms of pseudohyphae superficially resemble hyphae but have constrictions at the positions of septa (arrows) and show regular branching. Growth conditions were as follows: (a) YEPD pH6.0 at 308C; (b, c) YEPD pH6 plus 20% serum, grown at 378C; (d–f) YEPD pH 6.0 at 358C. The images in (f) and (g) were taken from the same culture. (h) Shown is an hsl1D/hsl1D mutant growing in YEPD at 308C. The images in (d) and (h) are of cells stained with Calcofluor white, which stains chitin in the cell walls and septa. All scale bars represent 10 mm (this figure is reproduced with permission from Sudbery et al. [84]).

Most of the fungal pathogens can primarily proliferate either in the form of budding yeast (e.g., *Cryptococcus neoformans*) or as a filamentous hyphal structure (e.g., *Aspergillus *spp.). However, *C. albicans* has a unique ability to grow in a variety of morphological forms and at least, four kinds of forms, that is, yeast-like, hyphae, pseudohyphae, and chlamydospores have been well documented [5, 80–83]. Among these morphological forms, the yeast-hyphae switch has been extensively studied due to its correlation with pathogenicity (Figure 1) [84]. In the 1950s, it was believed that hyphae rather than yeast form is responsible for pathogenic character of this organism. This was substantiated by the observation that *C. albicans, *injected into mice subcutaneously or intraperitoneally, was able to produce filamentous form within 1 hour of injection [85, 86]. Besides, it is quite amazing that the cell fate does not lock into a particular morphology rather it appears to be reversible and cells can be induced to form hyphae from their established buds [87].The ability to switch between different morphologies in response to a variety of environmental stimuli might have an important consequence for its survival in different conditions. For example, during human infections or growth on media containing blood serum at 37°C, hyphal cells are produced from budding yeast cells, the morphology of which could facilitate deep penetration of this pathogen into epithelia, endothelia, and human tissues [88]. Now the question is how this switch works and how it plays a subtle but significant role in virulence. Initially, it was observed that the mutations in two transcription factors, Cph1p and Efg1p, blocked the hyphal transition and also reduced the virulence [89]. This observation suggested that morphological switching ability plays an important role in virulence. Subsequently, a large number of transcription factors have been identified, and they are implicated in the yeast-hyphae shift and their functions have been analyzed with respect to virulence [90].

Another important aspect in this regard is the functioning of signaling pathways that modulate the network of transcription factors responsible for yeast-hyphae switch in response to extracelluar stimuli such as, presence of serum or N-acetyl-glucosamine in the media along with rise in temperature to 37°C [91, 92]. Usually the signals are transmitted from media to transcriptional machinery through a series of molecular cascades that affect the expression of large number of genes, which will subsequently change the morphology. Several signaling pathways that regulate the morphogenesis of *C. albicans* have been identified. The cAMP-dependent protein kinase pathway has been prominent among them. The cAMP-dependent pathway is responsible for regulation of Efg1p transcription factor, which is considered to be a master molecule for the general control of morphogenesis in *C. albicans* [93]. The regulation of Efg1p is thought to be through direct phosphorylation of this transcription factor by cAMP-dependent protein kinase [94]. Efg1p controls several morphogenetic processes such as the regulation of yeast-hyphae transition, generation of chlamydospore, and also the determination of cell shape during white-opaque switching [89, 95–97]. The cAMP-mediated signaling pathway has been extensively studied in relation to yeast-hyphae shift, and mutations in this pathway severely affect the hyphal development [98–102]. Other pathways that transmit external signals to the transcriptional machinery including pH-sensing pathway, matrix-sensing pathway, and MAP kinase signaling pathway have been implicated in the hyphal development as well. The transcription factor Cph1p is thought to be the target of MAP kinase pathway and implicated in yeast-hyphae transition. This was inferred from the observation that the double mutant *cph1efg1* of *C. albicans* is defective in filamentous growth, unable to form hyphae and pseudohyphae in response to external stimuli such as serum or macrophages. This double mutant is basically locked in yeast form and is avirulent in mouse model [89]. In another study, it has been shown that deletion in any of the four elements such as Cph1p and its three upstream kinases Cst20p, Hst7p, and Cek1p, involved in Cph1p regulation, does not block the filament formation by serum and there is no dramatic changes in the transcription profile of the yeast-hyphal transition. This observation suggests that Cph1p is helpful but not essential for filament induction [103]. Furthermore, it has been shown that the external pH plays an important role in the yeast-hyphae transition. The main transcription factor in this pathway is Rim101p, which is proteolytically activated by Rim13p, and loss of Rim101p function results in the block of alkaline-induced hyphal development [104, 105].

Though morphogenetic changes have been studied thoroughly and implicated in pathogenicity, recent observation made by Noble et al. have challenged this concept and decoupled morphogenetic switching and pathogenicity [106]. Perhaps, the extensive use of *URA3* selectable marker for generating the mutants and their subsequent analysis has complicated the understanding of virulence in *C. albicans*. Several studies have clearly established that *URA3* plays a significant role in normal virulence as well as in yeast-hyphae transition [107–111]. The phenotypes of the mutants generated by using *URA3* selectable marker in this regard are questionable as the expression of *URA3* would vary greatly depending upon its chromosomal locations [107–109]. To circumvent the above-mentioned problem, Noble et al. have used a different set of selectable markers (*Candida dubliniensis HIS1 and Candida maltosa LEU2*), which are neutral for virulence, for homozygous disruption of about 11% of total genes of *C. albicans,* and found that there is no correlation between morphogenetic forms and virulence [106, 112]. Rather, it has been suggested that the biosynthetic pathway for glucosylceramide plays an important role in virulence, at least in mouse model [106].

## 5. Biofilm Formation and Drug Resistance

One of the significantly important attributes of *C. albicans* is the formation of biofilm on solid surfaces such as dental enamel and human heart valves in a three-dimensional fashion [113, 114]. From human health point of view, biofilms are important because they are developed on implanted medical devices, and it contributes to about half of all nosocomial infections [115]. Formation of biofilm occurs in a systematic manner. For instance, the budding yeast cells are attached to surfaces and grow horizontally to form the basal layer. Subsequently, hyphal cells are produced and form the upper layer. Then, with further secretion, biofilms will be covered by extracellular matrix which is mainly composed of carbohydrates and proteins [114, 116, 117]. Efforts have been made to unravel the differences between *Candida* biofilm and free-living planktonic cells by understanding the gene regulatory network [118–121]. Gene regulatory networking functions through multiple transcription factors, which generally bind to cis-regulatory DNA elements of the target genes or interact with protein complex assembled onto it. A number of transcription factors including Efg1p, Cph1p, Efh1p, Rap1p, Ino4p, and Tec1p have been identified and they are implicated in the regulation of biofilm formation [119–121]. Though morphogenetic forms have been implicated in pathogenicity, as such biofilm formation does not depend upon any particular morphological forms. Rather, the mutants unable to form hyphae or yeast are able to develop biofilm [122].

One of the most important manifestations of biofilm formation is their high-level drug resistance to different antifungal drugs [114, 123, 124]. It has also been demonstrated that individual cells disrupted from biofilms are more resistant to the available drugs than the free-living planktonic cells [125–127]. This suggests that the biofilm formation perhaps affects other factors in the cell, and these changes made during biofilm formation remain active even after the dissociation of the individual cells from biofilms. This drug resistance has certainly affected the whole scenario of management of *Candida*-related infections and complicates the treatment of *Candida*-infected patients [121, 128–130]. It was postulated that this high drug resistance could be due to low penetration of drugs through the biofilm. However, this idea does not hold true as mutants having defective biofilm can also show high-level of drug resistance [131, 132].

The drug resistance of *C. albicans* is posing a daunting challenge to *Candida* community and its understanding lies in unraveling different parameters that are responsible for this phenomenon. Rigorous efforts are being made in this regard, and several mechanisms have been proposed to be working for the drug resistance in this pathogen [133, 134]. For example, *C. albicans* is getting resistant to many antifungal drugs like flucytosine, fluconazole, amphotericin B, and caspofungin that are commonly used to treat fungal infections. Several studies have shown that mutations which affect uptake of flucytosine or its intracellular conversion are found to be major causes of resistance to these drugs [135–139]. Mutations in the gene *ERG11*, that encodes lanosterol 14*α*-demethylase, can affect the binding of azole drugs to this enzyme resulting in the increased drug resistance of the cells [140–142]. Also the overexpression of the gene *ERG11* and other *ERG* genes involved in ergosterol biosynthetic pathway increases resistance to drugs. The overexpression of these genes is mainly caused by an activating mutation in their regulator Upc2 [143]. Furthermore, in *C. albicans*, multiple drug resistance is mediated by two types of efflux pumps, ATP-binding cassette transporters and major facilitators. This subject has been extensively reviewed [134, 144, 145]. 

## 6. *Candida albicans *Genome

The *Candida albicans* strain SC5314 was taken for complete genome sequencing due to its wide spread use for molecular and genetic analysis worldwide [146, 147]. Generally, the genome of *C. albicans* is quite dynamic and lot of truncations, translocations, and other mutational events occur more frequently compared to other microbes. However, the genome of sequencing strain SC5314 was found to be quite stable having eight distinct chromosomes in duplicate ranging from 1030 to 3200 kb. The genomic sequence of this pathogen was published in 2004 as 266 independent contigs across a haploid reference genome and subsequently, a chromosome-level assembly was constructed [20, 148]. The genome size of *C. albicans* is estimated to be 14.3 Mb, and it contains about 6107 protein-coding genes [149]. Of the 6107 genes/ORFs, about 774 are specific to *C. albicans* and homologues for these genes/ORFs are not available in *S. cerevisiae* or *S. pombe* [20, 150]. Effort has been made to sequence the genomes of other less infectious *Candida* strains, and the complete genome sequences for *C. albicans *(WO-1), *C. dubliniensis, C. tropicalis, C. parapsilosis*, *Candida guilliermondii,* and *Candida lusitaniae* are available now [149]. The *Candida *Genome Database (CGD) is constantly updating the information pertaining to functions of unknown ORFs and other genes [151–154]. Moreover, a large number of genomic resources have been made and several of them are widely available to the research community (e.g., CandidaDB at http://genodb.pasteur.fr/cgi-bin/WebObjects/CandidaDB; Genolevures at http://www.genolevures.org/yeastgenomes.html Sanger Institute at http://www.sanger.ac.uk/resources/downloads/fungi/ and Broad Fungal Genome Initiative at http://www.broadinstitute.org/scientific-community/science/projects/fungal-genome-initiative/fungal-genome-initiative) [153]. Gene annotation data available in CGD shows that the functions of only 22.97% (1403 genes) of the genes have been experimentally verified, whereas 77.03% (4705 genes) of the genes remain uncharacterized in *C. albicans* and their functions have been assigned on the basis of sequence analysis. Furthermore, 152 genes/ORFs are still in the “dubious” category for which no experimental evidence is available and seems to be indistinguishable from noncoding sequences [154].

## 7. *Candida albicans* as a Fungal Model

Though *C. albicans* has been known to humans for quite long time, it did not catch the attention of scientific community, especially the fungal geneticists, till late 1960s and it was not studied vigorously till 1990s. Now the question is why there is so much delay. It is not simply possible to answer this question directly, but apparently the reason could be the sudden increase in immunocompromised patients due to the change in modern medical techniques in the 1970s and 1980s. These patients were infected by a variety of microbes, and *Candida* species were found to be one of the major pathogens [155–157]. Around this time, many well-known laboratories started working on this pathogen along with budding yeast *S. cerevisiae*. 

Initially, *C. albicans* was studied mostly from genetics point of view as molecular biology techniques were not available for this [158]. Furthermore, due to the non-availability of haploid version (as discussed above), it was not amenable to conventional genetic approaches and suffered a great setback. Besides, *C. albicans* genome was found to be quite unstable and heterozygous and hampered its study. But 1990s witnessed the exponential growth of research activities on *C. albicans* due to the availability of modern molecular biology, genomics and proteomics techniques. As other model organisms were getting sequenced, *Candida* community took the initiative of sequencing the genome of *C. albicans* strain SC5314, a clinical isolate, in 1996 funded by the Burroughs Wellcome Fund and the National Institute of Craniofacial and Dental Research and completed in 2004 [20, 146, 159]. The complete genome sequence has been extremely useful to address many fundamental questions. In the last two decades, an-ever increasing number of yeast geneticists and molecular biologists have taken up *C. albicans* as their primary research system. Many derivate from parental strain SC5314 have been made with a large number of markers paving the way for disruption of both the alleles without much difficulty to analyze the functions of the genes including their role in virulence [112, 147, 160–162]. In short, *C. albicans* has almost all the features of the model budding yeast *S. cerevisiae* like dispersed cells, replica plating, rapid growth, DNA transformation system, and gene disruption but still maintains its pathogenic character. Among the fungi, it is second only to *S. cerevisiae* and first among the pathogenic fungi as far as understanding of molecular biology, genetics, and pathogenicity is concerned. Therefore, in the last two decades, it has clearly emerged as a model for studying fungal pathogens and its complete understanding will shed light into virulence characters of other pathogenic fungi as well. 

## 8. Pathogenicity of *Candida albicans*

The pathogenicity of *C. albicans* depends upon two major factors. One is the immune status of the host and another is related to the virulence factors of this pathogen. In the last three decades, several laboratories have identified a large number of virulence factors of this important pathogen, and they have been implicated in the pathogenesis. Broadly, the microbial factors contributing to pathogenicity are the adhesion to the host cell, secretion of hydrolytic enzymes, dimorphic phenotype (yeast to filamentous form or hyphae), phenotypic switching, modulating host's immune system and formation of biofilm on biotic and abiotic surfaces [7, 163–167]. Adhesion is thought to be an essential step for colonization and establishment of *Candida* infections. *C. albicans* is a very versatile pathogen and has the ability to adhere to a variety of surfaces like endothelial cells, implanted inert materials in the host body, extracellular matrix, and epithelial cells. For adherence, it uses multiple mechanisms and they are well documented in the literature [168–175]. The molecules that are responsible for adhesion are called adhesins, which include Als1p-Als7p and Asl9p, Hwp1p, Int1p, Mnt1p, and several others [176–178].

*C. albicans* possesses an array of secreted hydrolytic enzymes of which SAPs (secreted aspartyl proteinases) are considered to be virulence factors and they contribute to the pathogenesis of candidiasis. The role of these SAPs in pathogenicity has been deduced from the observations that they are secreted *in vivo* during infection, the enzymes have the ability to degrade a number of important host factors, and the mutant strains have reduced virulence [167, 179, 180]. Moreover, *C. albicans *also secretes phospholipases A, B, and C, and they are considered to be putative virulence factors. These enzymes are associated with the function related to host cell damage, adherence, and penetration [7, 181].

 Another important feature of *C. albicans* is its ability to form biofilm on biotic and abiotic surfaces (as discussed above),and it is thought to be playing an important role as far as its pathogenicity is concerned [182, 183]. Phenotypic switching of *C. albicans* is considered to be one important virulence determinant. A panel of colony types like rough, smooth, irregular wrinkled and fuzzy, star, hat and stippled have been found for this pathogen [184, 185]. Among the phenotype switching, white-opaque switching of the clinical isolate, WO-1, is well studied [74]. Besides, *C. albicans* has the ability to grow as unicellular budding yeast or filamentous form of hyphae or pseudohyphae [186]. Altogether,* C. albicans *has the plasticity in phenotypic or morphological states, and could play an important role in pathogenicity, however, much more rigorous studies need to be carried out to have thorough understanding of the relationships between different morphogenetic states and their role in pathogenesis.

## 9. Two Other Fungal Pathogenic Models 

Two other fungal models which are extensively studied are *Cryptococcus neoforman *and *Aspergillus fumigatus*. They are briefly discussed below. 

### 9.1. *Cryptococcus Neoformans*

The basidiomycetous yeast *Cryptococcus neoformans* is one of the prominent fungal pathogens and responsible for morbidity and mortality in immunocompromised individuals including AIDS patients and people undergoing immunosuppressive therapy [187]. This organism exists primarily as haploid in nature and comes in contact with humans much more frequently. The immunocompetent individuals are able to control and contain the infection and do not develop cryptococcosis. However, in immunocompromised patients, *C. neoformans* can cross the blood-brain barrier and infect the brain, which leads to the development of meningitis [188–190]. *C. neoformans* possesses a number of traits, which are directly correlated with its virulence. Among the well-established factors responsible for virulence in this pathogen are the production of polysaccharide capsule and ability to synthesize melanin. It has been shown that capsule formation has antiphagocytic and immunomodulatory functions, whereas melanin is implicated in the protection of this yeast from a variety of environmental and host's toxic factors [191–194]. 

Generally, *C. neoformans* is acquired by human beings through inhalation of spores or yeast into the lungs. For this reason, alveolar macrophages are proposed to be first line of defense against cryptococcosis. In fact, experimental evidence suggests that macrophages play a very important role in host defense against infection caused by this pathogen [195, 196]. On the other hand, several studies have shown that macrophages cannot engulf *C. neoformans* in absence of opsonizing agents such as complement or antibodies even after coincubation of more than 24 hours [191, 197, 198]. 

### 9.2. *Aspergillus fumigatus*

Another important fungus is *Aspergillus fumigatus, *which is unique among the microbes in the sense that it is a primary and opportunistic pathogen as well as a major allergen [199–201]. *A. fumigatus* forms airborne spores, which are known as conidia are normally inhaled by every human being. However, in immune competent hosts, the conidia are killed and cleared whereas in immunocompromised individuals, *A. fumigatus* can cause aspergillosis leading to mortality rates of about 50% in certain high-risk groups such as leukemia patients [200, 202]. This fungus is known to reproduce by asexual means; however, compelling evidence has been gathered in the last one decade for the presence of mating-type genes and the expression of sex-related genes [203, 204]. Subsequently, it has been established that this fungus is equipped with a fully functional sexual reproductive cycle, which leads to the production of cleistothecia and ascospores [205–207]. The pathogenicity of *A. fumigatus* depends upon multiple factors of both the host and the pathogen. For establishment of invasive diseases, the fungus uses the expression of multiple genes in a highly coordinated and sequential manner. These include the gene products for cell wall assembly, conidial germination, hyphal growth, resistance to oxidative stress, and nutrient acquisition [208, 209].

Over the last two decades, a large number of fungi have got their genome sequenced including important fungal pathogens like *C. albicans*, *A. fumigatus*, *C. neoformans,* and others [20, 210, 211]. This has undoubtedly opened enormous avenues to have comparative study of the genomes to identify the potential candidate genes responsible for virulence [212, 213]. The genome sizes of *C. albicans*, *A. fumigatus* and *C. neoformans* are 14.3 Mb, 19 Mb and 29 Mb respectively. Genome annotation and comparison with related species shows that *C. albicans* has about 700 *Candida*-specific genes, whereas *A. fumigatus* has 500 *fumigatus*-specific genes and *C. neoformans* has about 650 species specific genes. Due to these specific genes, the mode of infection and pathogenicity varies, and they need to be studied separately to understand their biology as well as their ability to infect and propagate diseases. 

## 10. Conclusion

*C. albicans* has been of great interest to the scientific community for its pathogenic nature, and it is infecting the ever-increasing immunocompromised patients worldwide. The genetics of this fungal pathogen is quite complex compared to the baker's yeast *S. cerevisiae,* and “classical genetics” has suffered a great setback in studying this organism. However, the availability of complete genome sequence has opened enormous amount of opportunity for *Candida* community to study it by applying “reverse genetics” approach using advanced molecular genetics technology, proteomics, and genomics tools. The sequencing of other *Candida* species along with *C. albicans* has provided an opportunity to compare the genetic profile of these organisms and find out potential genes whose products are involved in adhesion, propagation, colonization, and survival in different niches in human and animal bodies. 

Moreover, *C. albicans* possesses most of the characteristics of *S. cerevisiae* and more than 80% genes are similar in both the organisms. It is also easy to handle in the laboratory and can grow on standard yeast media without much problem. Of late, many yeast researchers prefer working on *C. albicans* rather than *S.cerevisiae* as lot of cellular mechanisms in *Candida *are still unknown and discovery of them could be quite interesting. In summary, *C. albicans* has catapulted into the centre stage of study for fungal pathogens, and clearly, it has emerged as one of the models for fungal pathogens. 

## 11. Future Perspective

Though *C. albicans* has got lot of attention from scientific community, understanding the complete life style is far from over. The genome sequencing of this pathogen has revealed about 6107 genes but most of the functions assigned to them are borrowed from other organisms through sequence similarity. In another ten years or so, we have to assign the functions to each of the gene by experimental evidence so that it can serve as a “gold standard” as it is done for *S. cerevisiae*. Special attention must be given to *Candida*-specific genes, which account for about 13% of the total genes for which there is no homologues in *S. cerevisiae*. This will certainly shed light on understanding the pathogenic nature of this organism. Furthermore, to test different mutants, better mouse models can be developed so that all the genes responsible for virulence can be identified. It has been all the more important in the light of the recent findings that there is hardly any correlation between morphogenetic forms and its pathogenicity. As *Candida* species are becoming resistant to available drugs, efforts must be made to develop potential antifungal drugs. As many immunocompromised patients cannot tolerate high doses of antifungal drugs due to their side effects, biomedicines must be developed to treat the patients. In future, it will be necessary to have genetic and biochemical networks of all the pathways responsible for biofilm formation, drug resistance, yeast-hyphae transition, white-opaque switch, and their interactions with each other. This will help to develop avirulent strains in true sense, which can be locked in commensal state and can be used for vaccination to augment the immune system. Revealing the mechanism of pathogenicity in this organism will be useful in handing other fungal pathogens, and it will serve as a reference strain for them. 

## References

[1] Ruhnke M, Maschmeyer G (2002). Management of mycoses in patients with hematologic disease and cancer—review of the literature. *European Journal of Medical Research*.

[2] Meiller TF, Hube B, Schild L (2009). A novel immune evasion strategy of *Candida albicans*: proteolytic cleavage of a salivary antimicrobial peptide. *PLoS One*.

[3] Schulze J, Sonnenborn U (2009). Yeast in the Gut: from commensals to infectious agents. *Deutsches Arzteblatt*.

[4] Sobel JD (1997). Vaginitis. *New England Journal of Medicine*.

[5] Odds FC (1988). *Candida and Candidosis*.

[6] Soll DR, Staebell M, Langtimm C, Pfaller M, Hicks J, Rao TVG (1988). Multiple *Candida* strains in the course of a single systemic infection. *Journal of Clinical Microbiology*.

[7] Calderone RA (2002). *Candida and Candidiasis*.

[8] Sexton JA, Brown V, Johnston M (2007). Regulation of sugar transport and metabolism by the *Candida albicans* Rgt1 transcriptional repressor. *Yeast*.

[9] Pfaller MA, Diekema DJ (2007). Epidemiology of invasive candidiasis: a persistent public health problem. *Clinical Microbiology Reviews*.

[10] Schelenz S (2008). Management of candidiasis in the intensive care unit. *The Journal of Antimicrobial Chemotherapy*.

[11] Gozalbo D, Roig P, Villamón E, Gil ML (2004). *Candida* and candidiasis: the cell wall as a potential molecular target for antifungal therapy. *Current Drug Targets*.

[12] Anderson ML, Odds FC (1985). Adherence of *Candida albicans* to vaginal epithelia: significance of morphological form and effect of ketoconazole. *Mykosen*.

[13] Gordon MA (1958). Rapid serological differentiation of *Candida albicans* from *Candida* stellatoidea. *Journal of Investigative Dermatology*.

[14] GORDON MA (1958). Differentiation of yeasts by means of fluorescent antibody. *Proceedings of the Society for Experimental Biology and Medicine*.

[15] Taschdjian CL, Burchall JJ, Kozinn PJ (1960). Rapid identification of *Candida albicans* by filamentation on serum and serum substitutes. *A. M. A. Journal of Diseases of Children*.

[16] Gudlaugsson O, Gillespie S, Lee K (2003). Attributable mortality of nosocomial candidemia, revisited. *Clinical Infectious Diseases*.

[17] Eggimann P, Garbino J, Pittet D (2003). Epidemiology of *Candida* species infections in critically ill non-immunosuppressed patients. *Lancet Infectious Diseases*.

[18] Wey SB, Mori M, Pfaller MA, Woolson RF, Wenzel RP (1988). Hospital-acquired candidemia. The attributable mortality and excess length of stay. *Archives of Internal Medicine*.

[19] Patel SJ, Saiman L (2010). Antibiotic resistance in neonatal intensive care unit pathogens: mechanisms, clinical impact, and prevention including antibiotic stewardship. *Clinics in Perinatology*.

[20] Jones T, Federspiel NA, Chibana H (2004). The diploid genome sequence of *Candida albicans*. *Proceedings of the National Academy of Sciences of the United States of America*.

[21] Wang Z, Gerstein M, Snyder M (2009). RNA-Seq: a revolutionary tool for transcriptomics. *Nature Reviews Genetics*.

[22] Nagalakshmi U, Wang Z, Waern K (2008). The transcriptional landscape of the yeast genome defined by RNA sequencing. *Science*.

[23] Wilhelm BT, Marguerat S, Watt S (2008). Dynamic repertoire of a eukaryotic transcriptome surveyed at single-nucleotide resolution. *Nature*.

[24] Cloonan N, Forrest ARR, Kolle G (2008). Stem cell transcriptome profiling via massive-scale mRNA sequencing. *Nature Methods*.

[25] Emrich SJ, Barbazuk WB, Li L, Schnable PS (2007). Gene discovery and annotation using LCM-454 transcriptome sequencing. *Genome Research*.

[26] Vera JC, Wheat CW, Fescemyer HW (2008). Rapid transcriptome characterization for a nonmodel organism using 454 pyrosequencing. *Molecular Ecology*.

[27] Su Z, Ning B, Fang H (2011). Next-generation sequencing and its applications in molecular diagnostics. *Expert Review of Molecular Diagnostics*.

[28] Kapranov P, Ozsolak F, Milos PM (2012). Profiling of short RNAs using helicos single-molecule sequencing. *Methods in Molecular Biology*.

[29] Busby MA, Gray JM, Costa AM (2011). Expression divergence measured by transcriptome sequencing of four yeast species. *BMC Genomics*.

[30] Croucher NJ, Fookes MC, Perkins TT (2009). A simple method for directional transcriptome sequencing using illumina technology. *Nucleic Acids Research*.

[31] Lister R, O’Malley RC, Tonti-Filippini J (2008). Highly integrated single-base resolution maps of the epigenome in arabidopsis. *Cell*.

[32] Hughes ME, Grant GR, Paquin C, Qian J, Nitabach MN (2012). Deep sequencing the circadian and diurnal transcriptome of Drosophila brain. *Genome Research*.

[33] Li SM, Valo Z, Wang J, Gao H, Bowers CW, Singer-Sam J (2012). Transcriptome-wide survey of mouse CNS-Derived cells reveals monoallelic expression within novel gene families. *PLoS One*.

[34] Kalyana-Sundaram S, Kumar-Sinha C, Shankar S (2012). Expressed pseudogenes in the transcriptional landscape of human cancers. *Cell*.

[35] Bruno VM, Wang Z, Marjani SL (2010). Comprehensive annotation of the transcriptome of the human fungal pathogen *Candida albicans* using RNA-seq. *Genome Research*.

[36] Mitrovich QM, Tuch BB, de la Vega FM, Guthrie C, Johnson AD (2010). Evolution of yeast noncoding RNAs reveals an alternative mechanism for widespread intron loss. *Science*.

[37] Gilmour DS, Lis JT (1984). Detecting protein-DNA interactions *in vivo*: distribution of RNA polymerase on specific bacterial genes. *Proceedings of the National Academy of Sciences of the United States of America*.

[38] Gilmour DS, Lis JT (1985). *In vivo* interactions of RNA polymerase II with genes of *Drosophila melanogaster*. *Molecular and Cellular Biology*.

[39] Gilmour DS, Lis JT (1986). RNA polymerase II interacts with the promoter region of the noninduced hsp70 gene in *Drosophila melanogaster* cells. *Molecular and Cellular Biology*.

[40] Solomon MJ, Larsen PL, Varshavsky A (1988). Mapping protein-DNA interactions *in vivo* with formaldehyde: svidence that histone H4 is retained on a highly transcribed gene. *Cell*.

[41] Collas P (2010). The current state of chromatin immunoprecipitation. *Molecular Biotechnology*.

[42] Kim TH, Ren B (2006). Genome-wide analysis of protein-DNA interactions. *Annual Review of Genomics and Human Genetics*.

[43] Lickwar CR, Mueller F, Hanlon SE, McNally JG, Lieb JD (2012). Genome-wide protein-DNA binding dynamics suggest a molecular clutch for transcription factor function. *Nature*.

[44] Davis SE, Mooney RA, Kanin EI, Grass J, Landick R, Ansari AZ (2011). Mapping *E. coli* RNA polymerase and associated transcription factors and identifying promoters genome-wide. *Methods in Enzymology*.

[45] Barski A, Cuddapah S, Cui K (2007). High-resolution profiling of histone methylations in the human genome. *Cell*.

[46] Loh YH, Wu Q, Chew JL (2006). The Oct4 and Nanog transcription network regulates pluripotency in mouse embryonic stem cells. *Nature Genetics*.

[47] Wei CL, Wu Q, Vega VB (2006). A global map of p53 transcription-factor binding sites in the human genome. *Cell*.

[48] Lee TI, Johnstone SE, Young RA (2006). Chromatin immunoprecipitation and microarray-based analysis of protein location. *Nature Protocols*.

[49] Hanlon SE, Lieb JD (2004). Progress and challenges in profiling the dynamics of chromatin and transcription factor binding with DNA microarrays. *Current Opinion in Genetics and Development*.

[50] Sikder D, Kodadek T (2005). Genomic studies of transcription factor-DNA interactions. *Current Opinion in Chemical Biology*.

[51] Sellam A, Tebbji F, Nantel A (2009). Role of Ndt80p in sterol metabolism regulation and azole resistance in *Candida albicans*. *Eukaryotic Cell*.

[52] Lassak T, Schneider E, Bussmann M (2011). Target specificity of the *Candida albicans* Efg1 regulator. *Molecular Microbiology*.

[53] Znaidi S, Barker KS, Weber S (2009). Identification of the *Candida albicans* Cap1p regulon. *Eukaryotic Cell*.

[54] Liu TT, Znaidi S, Barker KS (2007). Genome-wide expression and location analyses of the *Candida albicans* Tac1p regulon. *Eukaryotic Cell*.

[55] Bish JT, Sarachek A (1967). Influences of temperature and adenine concentration upon the cultural instability of a red adenine auxotroph of *Candida albicans*. *Mycologia*.

[56] Whelan WL, Partridge RM, Magee PT (1980). Heterozygosity and segregation in *Candida albicans*. *Molecular and General Genetics*.

[57] Whelan WL, Magee PT (1981). Natural heterozygosity in *Candida albicans*. *Journal of Bacteriology*.

[58] Whelan WL, Soll DR (1982). Mitotic recombination in *Candida albicans*: recessive lethal alleles linked to a gene required for methionine biosynthesis. *Molecular and General Genetics*.

[59] Poulter R, Hanrahan V, Jeffery K (1982). Recombination analysis of naturally diploid *Candida albicans*. *Journal of Bacteriology*.

[60] Poulter RTM, Rikkerink EHA (1983). Genetic analysis of red, adenine-requiring mutants of *Candida albicans*. *Journal of Bacteriology*.

[61] Riggsby WS, Torres-Bauza LJ, Wills JW, Townes TM (1982). DNA content, kinetic complexity, and the ploidy question in *Candida albicans*. *Molecular and Cellular Biology*.

[62] Olaiya AF, Sogin SJ (1979). Ploidy determination of *Candida albicans*. *Journal of Bacteriology*.

[63] Larriba G, Calderone RA, San-Blas G, Calderone RA (2008). Heterozygosity and loss of heterozygosity in *Candida albicans*. *Pathogenic Fungi: Insights in Molecular Biology*.

[64] Poulter R, Jeffery K, Hubbard MJ (1981). Parasexual genetic analysis of *Candida albicans* by spheroplast fusion. *Journal of Bacteriology*.

[65] Sarachek A, Rhoads DD, Schwarzhoff RH (1981). Hybridization of *Candida albicans* through fusion of protoplasts. *Archives of Microbiology*.

[66] Barnett JA (2007). A history of research on yeast 10: foundations of yeast genetics. *Yeast*.

[67] Hull CM, Johnson AD (1999). Identification of a mating type-like locus in the asexual pathogenic yeast *Candida albicans*. *Science*.

[68] Bennett RJ, Johnson AD (2003). Completion of a parasexual cycle in *Candida albicans* by induced chromosome loss in tetraploid strains. *The EMBO Journal*.

[69] Madhani HD (2006). *From A to Alpha: Yeast as a Model for Cellular Differentiation*.

[70] Magee BB, Magee PT (2000). Induction of mating in *Candida albicans* by construction of *MTLa* and *MTLalpha* strains. *Science*.

[71] Hull CM, Raisner RM, Johnson AD (2000). Evidence for mating of the “asexual” yeast *Candida albicans* in a mammalian host. *Science*.

[72] Bennett RJ, Johnson AD (2005). Mating in *Candida albicans* and the search for a sexual cycle. *Annual Review of Microbiology*.

[73] Noble SM, Johnson AD (2007). Genetics of *Candida albicans*, a diploid human fungal pathogen. *Annual Review of Genetics*.

[74] Slutsky B, Staebell M, Anderson J, Pfaller M, Soll DR (1987). ‘White-opaque transition’: a second high-frequency switching system in *Candida albicans*. *Journal of Bacteriology*.

[75] Miller MG, Johnson AD (2002). White-opaque switching in *Candida albicans* is controlled by mating-type locus homeodomain proteins and allows efficient mating. *Cell*.

[76] Rustchenko-Bulgac EP, Sherman F, Hicks JB (1990). Chromosomal rearrangements associated with morphological mutants provide a means for genetic variation of *Candida albicans*. *Journal of Bacteriology*.

[77] Rustchenko EP, Howard DH, Sherman F (1994). Chromosomal alterations of *Candida albicans* are associated with the gain and loss of assimilating functions. *Journal of Bacteriology*.

[78] Janbon G, Sherman F, Rustchenko E (1998). Monosomy of a specific chromosome determines L-sorbose utilization: a novel regulatory mechanism in *Candida albicans*. *Proceedings of the National Academy of Sciences of the United States of America*.

[79] Selmecki A, Forche A, Berman J (2006). Aneuploidy and isochromosome formation in drug-resistant *Candida albicans*. *Science*.

[80] Merson-Davies LA, Odds FC (1989). A morphology index for characterization of cell shape in *Candida albicans*. *Journal of General Microbiology*.

[81] Odds FC (1985). Morphogenesis in *Candida albicans*. *Critical reviews in microbiology*.

[82] Berman J, Sudbery PE (2002). *Candida albicans*: a molecular revolution built on lessons from budding yeast. *Nature Reviews Genetics*.

[83] Gow NA (1997). Germ tube growth of *Candida albicans*. *Current Topics in Medical Mycology*.

[84] Sudbery P, Gow N, Berman J (2004). The distinct morphogenic states of *Candida albicans*. *Trends in Microbiology*.

[85] Hill DW, Gebhardt LP (1956). Morphological transformation of *Candida albicans*in tissues of mice. *Proceedings of the Society For Experimental Biology and Medicine*.

[86] Young G (1958). The process of invasion and the persistence of *Candida albicans*injected intraperitoneally into mice. *Journal of Infectious Diseases*.

[87] Hazan I, Sepulveda-Becerra M, Liu H (2002). Hyphal elongation is regulated independently of cell cycle in *Candida albicans*. *Molecular Biology of the Cell*.

[88] Gow NAR, Brown AJP, Odds FC (2002). Fungal morphogenesis and host invasion. *Current Opinion in Microbiology*.

[89] Lo HJ, Köhler JR, Didomenico B, Loebenberg D, Cacciapuoti A, Fink GR (1997). Nonfilamentous C. albicans mutants are avirulent. *Cell*.

[90] Whiteway M, Bachewich C (2007). Morphogenesis in *Candida albicans*. *Annual Review of Microbiology*.

[91] Whiteway M, Oberholzer U (2004). *Candida* morphogenesis and host-pathogen interactions. *Current Opinion in Microbiology*.

[92] Mattia E, Carruba G, Angiolella L, Cassone A (1982). Induction of germ tube formation by N-acetyl-D-glucosamine in *Candida albicans*: uptake of inducer and germinative response. *Journal of Bacteriology*.

[93] Stoldt VR, Sonneborn A, Leuker CE, Ernst JF (1997). Efg1p, an essential regulator of morphogenesis of the human pathogen *Candida albicans*, is a member of a conserved class of bHLH proteins regulating morphogenetic processes in fungi. *The EMBO Journal*.

[94] Bockmüh DP, Ernst JF (2001). A potential phosphorylation site for an A-Type kinase in the Efgl regulator protein contributes to hyphal morphogenesis of *Candida albicans*. *Genetics*.

[95] Doedt T, Krishnamurthy S, Bockmühl DP (2004). APSES proteins regulate morphogenesis and metabolism in *Candida albicans*. *Molecular Biology of the Cell*.

[96] Sonneborn A, Bockmühl DP, Ernst JF (1999). Chlamydospore formation in *Candida albicans* requires the Efg1p morphogenetic regulator. *Infection and Immunity*.

[97] Srikantha T, Tsai LK, Daniels K, Soll DR (2000). EFG1 null mutants of *Candida albicans* switch but cannot express the complete phenotype of white-phase budding cells. *Journal of Bacteriology*.

[98] Bahn YS, Staab J, Sundstrom P (2003). Increased high-affinity phosphodiesterase *PDE2* gene expression in germ tubes counteracts CAP1-dependent synthesis of cyclic AMP, limits hypha production and promotes virulence of *Candida albicans*. *Molecular Microbiology*.

[99] Cloutier M, Castilla R, Bolduc N (2003). The two isoforms of the cAMP-dependent protein kinase catalytic subunit are involved in the control of dimorphism in the human fungal pathogen *Candida albicans*. *Fungal Genetics and Biology*.

[100] Jung WH, Stateva LI (2003). The cAMP phosphodiesterase encoded by *CaPDE2* is required for hyphal development in *Candida albicans*. *Microbiology*.

[101] Leberer E, Harcus D, Dignard D (2001). Ras links cellular morphogenesis to virulence by regulation of the MAP kinase and cAMP signalling pathways in the pathogenic fungus *Candida albicans*. *Molecular Microbiology*.

[102] Maidan MM, de Rop L, Serneels J (2005). The G protein-coupled receptor Gpr1 and the G*α* protein Gpa2 act through the cAMP-protein kinase A pathway to induce morphogenesis in *Candida albicans*. *Molecular Biology of the Cell*.

[103] Huang H, Harcus D, Whiteway M (2008). Transcript profiling of a MAP kinase pathway in *C. albicans*. *Microbiological Research*.

[104] Davis D, Wilson RB, Mitchell AP (2000). *RIM101*-dependent and -independent pathways govern pH responses in *Candida albicans*. *Molecular and Cellular Biology*.

[105] Li M, Martin SJ, Bruno VM, Mitchell AP, Davis DA (2004). *Candida albicans* Rim13p, a protease required for Rim101p processing at acidic and alkaline pHs. *Eukaryotic Cell*.

[106] Noble SM, French S, Kohn LA, Chen V, Johnson AD (2010). Systematic screens of a *Candida albicans* homozygous deletion library decouple morphogenetic switching and pathogenicity. *Nature Genetics*.

[107] Brand A, MacCallum DM, Brown AJP, Gow NAR, Odds FC (2004). Ectopic expression of *URA3* can influence the virulence phenotypes and proteome of *Candida albicans* but can be overcome by targeted reintegration of *URA3* at the *RPS10* locus. *Eukaryotic Cell*.

[108] Cheng S, Nguyen MH, Zhang Z, Jia H, Handfield M, Clancy CJ (2003). Evaluation of the roles of four *Candida albicans* genes in virulence by using gene disruption strains that express URA3 from the native locus. *Infection and Immunity*.

[109] Lay J, Henry LK, Clifford J, Koltin Y, Bulawa CE, Becker JM (1998). Altered expression of selectable marker *URA3* in gene-disrupted *Candida albicans* strains complicates interpretation of virulence studies. *Infection and Immunity*.

[110] Sharkey LL, Liao WL, Ghosh AK, Fonzi WA (2005). Flanking direct repeats of hisG alter *URA3* marker expression at the *HWP1* locus of *Candida albicans*. *Microbiology*.

[111] Sundstrom P, Cutler JE, Staab JF (2002). Reevaluation of the role of *HWP1* in systemic candidiasis by use of *Candida albicans* strains with selectable marker *URA3* targeted to the *ENO1* locus. *Infection and Immunity*.

[112] Noble SM, Johnson AD (2005). Strains and strategies for large-scale gene deletion studies of the diploid human fungal pathogen *Candida albicans*. *Eukaryotic Cell*.

[113] Lamfon H, Porter SR, McCullough M, Pratten J (2003). Formation of *Candida albicans* biofilms on non-shedding oral surfaces. *European Journal of Oral Sciences*.

[114] Douglas LJ (2003). *Candida* biofilms and their role in infection. *Trends in Microbiology*.

[115] Kojic EM, Darouiche RO (2004). *Candida* infections of medical devices. *Clinical Microbiology Reviews*.

[116] Chandra J, Kuhn DM, Mukherjee PK, Hoyer LL, McCormick T, Ghannoum MA (2001). Biofilm formation by the fungal pathogen *Candida albicans*: development, architecture, and drug resistance. *Journal of Bacteriology*.

[117] Baillie GS, Douglas LJ (2000). Matrix polymers of *Candida* biofilms and their possible role in biofilm resistance to antifungal agents. *Journal of Antimicrobial Chemotherapy*.

[118] Wang YC, Lan CY, Hsieh WP, Murillo LA, Agabian N, Chen BS (2010). Global screening of potential *Candida albicans* biofilm-related transcription factors via network comparison. *BMC Bioinformatics*.

[119] Nobile CJ, Nett JE, Hernday AD (2009). Biofilm matrix regulation by *Candida albicans* Zap1. *PLoS Biology*.

[120] Norice CT, Smith FJ, Solis N, Filler SG, Mitchell AP (2007). Requirement for *Candida albicans* Sun41 in biofilm formation and virulence. *Eukaryotic Cell*.

[121] Blankenship JR, Mitchell AP (2006). How to build a biofilm: a fungal perspective. *Current Opinion in Microbiology*.

[122] Baillie GS, Douglas JJ (1999). Role of dimorphism in the development of *Candida albicans* biofilms. *Journal of Medical Microbiology*.

[123] Donlan RM, Costerton JW (2002). Biofilms: survival mechanisms of clinically relevant microorganisms. *Clinical Microbiology Reviews*.

[124] Kumamoto CA (2002). *Candida* biofilms. *Current Opinion in Microbiology*.

[125] Baillie GS, Douglas LJ (1998). Effect of growth rate on resistance of *Candida albicans* biofilms to antifungal agents. *Antimicrobial Agents and Chemotherapy*.

[126] Baillie GS, Douglas LJ (1998). Iron-limited biofilms of *Candida albicans* and their susceptibility to amphotericin B. *Antimicrobial Agents and Chemotherapy*.

[127] Ramage G, Bachmann S, Patterson TF, Wickes BL, López-Ribot JL (2002). Investigation of multidrug efflux pumps in relation to fluconazole resistance in *Candida albicans* biofilms. *Journal of Antimicrobial Chemotherapy*.

[128] Lewis K (2008). Multidrug tolerance of biofilms and persister cells. *Current Topics in Microbiology and Immunology*.

[129] Mukherjee PK, Zhou G, Munyon R, Ghannoum MA (2005). *Candida* biofilm: a well-designed protected environment. *Medical Mycology*.

[130] Nobile CJ, Mitchell AP (2006). Genetics and genomics of *Candida albicans* biofilm formation. *Cellular Microbiology*.

[131] Al-Fattani MA, Douglas LJ (2004). Penetration of *Candida* biofilms by antifungal agents. *Antimicrobial Agents and Chemotherapy*.

[132] Ramage G, VandeWalle K, López-Ribot JL, Wickes BL (2002). The filamentation pathway controlled by the Efg1 regulator protein is required for normal biofilm formation and development in *Candida albicans*. *FEMS Microbiology Letters*.

[133] Cannon RD, Lamping E, Holmes AR (2007). *Candida albicans* drug resistance—another way to cope with stress. *Microbiology*.

[134] Morschhäuser J (2010). Regulation of multidrug resistance in pathogenic fungi. *Fungal Genetics and Biology*.

[135] Dodgson AR, Dodgson KJ, Pujol C, Pfaller MA, Soll DR (2004). Clade-specific flucytosine resistance is due to a single nucleotide change in the FUR1 gene of *Candida albicans*. *Antimicrobial Agents and Chemotherapy*.

[136] Florent M, Noël T, Ruprich-Robert G (2009). Nonsense and missense mutations in *FCY2* and *FCY1* genes are responsible for flucytosine resistance and flucytosine-fluconazole cross-resistance in clinical isolates of *Candida lusitaniae*. *Antimicrobial Agents and Chemotherapy*.

[137] Hope WW, Tabernero L, Denning DW, Anderson MJ (2004). Molecular mechanisms of primary resistance to flucytosine in *Candida albicans*. *Antimicrobial Agents and Chemotherapy*.

[138] Noël T, François F, Paumard P, Chastin C, Brèthes D, Villard J (2003). Flucytosine-fluconazole cross-resistance in purine-cytosine permease-deficient *Candida lusitaniae* clinical isolates: indirect evidence of a fluconazole uptake transporter. *Antimicrobial Agents and Chemotherapy*.

[139] Papon N, Noël T, Florent M (2007). Molecular mechanism of flucytosine resistance in *Candida lusitaniae*: contribution of the *FCY2*, *FCY1*, and *FUR1* genes to 5-fluorouracil and fluconazole cross-resistance. *Antimicrobial Agents and Chemotherapy*.

[140] Kelly SL, Lamb DC, Kelly DE (1999). Y132H substitution in *Candida albicans* sterol 14*α*-demethylase confers fluconazole resistance by preventing binding to haem. *FEMS Microbiology Letters*.

[141] Kelly SL, Lamb DC, Loeffler J, Einsele H, Kelly DE (1999). The G464S amino acid substitution in *Candida albicans* sterol 14*α*-demethylase causes fluconazole resistance in the clinic through reduced affinity. *Biochemical and Biophysical Research Communications*.

[142] Lamb DC, Kelly DE, White TC, Kelly SL (2000). The R467K amino acid substitution in *Candida albicans* sterol 14*α*- demethylase causes drug resistance through reduced affinity. *Antimicrobial Agents and Chemotherapy*.

[143] Dunkel N, Liu TT, Barker KS, Homayouni R, Morschhäuser J, Rogers PD (2008). A gain-of-function mutation in the transcription factor Upc2p causes upregulation of ergosterol biosynthesis genes and increased fluconazole resistance in a clinical *Candida albicans* isolate. *Eukaryotic Cell*.

[144] Prasad R, Gaur NA, Gaur M, Komath SS (2006). Efflux pumps in drug resistance of *Candida*. *Infectious Disorders*.

[145] Sanglard D, Coste A, Ferrari S (2009). Antifungal drug resistance mechanisms in fungal pathogens from the perspective of transcriptional gene regulation. *FEMS Yeast Research*.

[146] Gillum AM, Tsay EYH, Kirsch DR (1984). Isolation of the *Candida albicans* gene for orotidine-5’-phosphate decarboxylase by complementation of *S.cerevisiae* ura3 and *E. coli*  
*pyrF* mutations. *Molecular and General Genetics*.

[147] Fonzi WA, Irwin MY (1993). Isogenic strain construction and gene mapping in *Candida albicans*. *Genetics*.

[148] van het Hoog M, Rast TJ, Martchenko M (2007). Assembly of the *Candida albicans* genome into sixteen supercontigs aligned on the eight chromosomes. *Genome Biology*.

[149] Butler G, Rasmussen MD, Lin MF (2009). Evolution of pathogenicity and sexual reproduction in eight *Candida genome*s. *Nature*.

[150] Kabir MA, Hussain MA (2009). Human fungal pathogen *Candida albicans* in the postgenomic era: an overview. *Expert Review of Anti-Infective Therapy*.

[151] Skrzypek MS, Arnaud MB, Costanzo MC (2009). New tools at the *Candida genome* Database: biochemical pathways and full-text literature search. *Nucleic Acids Research*.

[152] Arnaud MB, Costanzo MC, Skrzypek MS (2007). Sequence resources at the *Candida genome* Database. *Nucleic Acids Research*.

[153] Arnaud MB, Costanzo MC, Shah P, Skrzypek MS, Sherlock G (2009). Gene Ontology and the annotation of pathogen genomes: the case of *Candida albicans*. *Trends in Microbiology*.

[154] Inglis DO, Arnaud MB, Binkley J (2012). The *Candida genome* database incorporates multiple *Candida* species: multispecies search and analysis tools with curated gene and protein information for *Candida albicans* and *Candida glabrata*. *Nucleic Acids Research*.

[155] Edwards JE, Lehrer RI, Stiehm ER (1978). Severe candidal infections. Clinical perspective, immune defense mechanisms, and current concepts of therapy. *Annals of Internal Medicine*.

[156] Lerner CW, Tapper ML (1984). Opportunistic infection complicating acquired immune deficiency syndrome. Clinical features of 25 cases. *Medicine*.

[157] Odds FC (1987). *Candida* infections: an overview. *Critical Reviews in Microbiology*.

[158] Scherer S, Magee PT (1990). Genetics of *Candida albicans*. *Microbiological Reviews*.

[159] Magee BB, Magee PT (2005). Recent advances in the genomic analysis of *Candida albicans*. *Revista Iberoamericana de Micologia*.

[160] Nobile CJ, Mitchell AP (2009). Large-scale gene disruption using the *UAU1* cassette. *Methods in Molecular Biology*.

[161] Bruno VM, Mitchell AP (2004). Large-scale gene function analysis in *Candida albicans*. *Trends in Microbiology*.

[162] Enloe B, Diamond A, Mitchell AP (2000). A single-transformation gene function test in diploid *Candida albicans*. *Journal of Bacteriology*.

[163] Cutler JE (1991). Putative virulence factors of *Candida albicans*. *Annual Review of Microbiology*.

[164] Chaffin WL, López-Ribot JL, Casanova M, Gozalbo D, Martínez JP (1998). Cell wall and secreted proteins of *Candida albicans*: identification, function, and expression. *Microbiology and Molecular Biology Reviews*.

[165] Martínez JP, Gil ML, López-Ribot JL, Chaffin WL (1998). Serologic response to cell wall mannoproteins and proteins of *Candida albicans*. *Clinical Microbiology Reviews*.

[166] Soll DR (1992). High-frequency switching in *Candida albicans*. *Clinical Microbiology Reviews*.

[167] Naglik JR, Challacombe SJ, Hube B (2003). *Candida albicans* secreted aspartyl proteinases in virulence and pathogenesis. *Microbiology and Molecular Biology Reviews*.

[168] Calderone RA, Braun PC (1991). Adherence and receptor relationships of *Candida albicans*. *Microbiological Reviews*.

[169] Calderone RA (1993). Molecular interactions at the interface of *Candida albicans* and host cells. *Archives of Medical Research*.

[170] Douglas LJ, Bennet JE, Hay RJ, Peterson PK (1992). Mannoprotein adhesions of *Candida albicans*. *New Strategies in Fungal Disease*.

[171] Douglas LJ (1995). Adhesin-receptor interactions in the attachement of *Candida albicans*to host epithelial cells. *Canadian Journal of Botany*.

[172] Fukazawa Y, Kagaya K (1997). Molecular bases of adhesion of *Candida albicans*. *Journal of Medical and Veterinary Mycology*.

[173] Hostetter MK (1994). Adhesins and ligands involved in the interaction of *Candida* spp. with epithelial and endothelial surfaces. *Clinical Microbiology Reviews*.

[174] Klotz SA, Penn RL (1987). Multiple mechanisms may contribute to the adherence of *Candida* yeasts to living cells. *Current Microbiology*.

[175] Pendrak ML, Klotz SA (1995). Adherence of *Candida albicans* to host cells. *FEMS Microbiology Letters*.

[176] Liu Y, Filler SG (2011). *Candida albicans* Als3, a multifunctional adhesin and invasin. *Eukaryotic Cell*.

[177] Sturtevant J, Calderone R (1997). *Candida albicans* adhesins: biochemical aspects and virulence. *Revista Iberoamericana de Micologia*.

[178] Calderone RA, Fonzi WA (2001). Virulence factors of *Candida albicans*. *Trends in Microbiology*.

[179] Hube B, Sanglard D, Odds FC (1997). Disruption of each of the secreted aspartyl proteinase genes SAP1, SAP2, and SAP3 of *Candida albicans* attenuates virulence. *Infection and Immunity*.

[180] Schaller M, Borelli C, Korting HC, Hube B (2005). Hydrolytic enzymes as virulence factors of *Candida albicans*. *Mycoses*.

[181] Ghannoum MA (2000). Potential role of phospholipases in virulence and fungal pathogenesis. *Clinical Microbiology Reviews*.

[182] Ganguly S, Mitchell AP (2011). Mucosal biofilms of *Candida albicans*. *Current Opinion in Microbiology*.

[183] Ramage G, Mowat E, Jones B, Williams C, Lopez-Ribot J (2009). Our Current Understanding of Fungal Biofilms Fungal biofilms Gordon Ramage. *Critical Reviews in Microbiology*.

[184] Pomés R, Gil C, Nombela C (1985). Genetic analysis of *Candida albicans* morphological mutants. *Journal of General Microbiology*.

[185] Slutsky B, Buffo J, Soll DR (1985). High-frequency switching of colony morphology in *Candida albicans*. *Science*.

[186] Sudbery PE (2011). Growth of *Candida albicans* hyphae. *Nature Reviews Microbiology*.

[187] Park BJ, Wannemuehler KA, Marston BJ, Govender N, Pappas PG, Chiller TM (2009). Estimation of the current global burden of cryptococcal meningitis among persons living with *HIV/AIDS*. *AIDS*.

[188] Chen LC, Goldman DL, Doering TL, Pirofski LA, Casadevall A (1999). Antibody response to *Cryptococcus neoformans* proteins in rodents and humans. *Infection and Immunity*.

[189] Goldman DL, Khine H, Abadi J (2001). Serologic evidence for *Cryptococcus neoformans* infection in early childhood. *Pediatrics*.

[190] Kim KS (2006). Microbial translocation of the blood-brain barrier. *International Journal for Parasitology*.

[191] Liu OW, Chun CD, Chow ED, Chen C, Madhani HD, Noble SM (2008). Systematic genetic analysis of virulence in the human fungal pathogen *Cryptococcus neoformans*. *Cell*.

[192] Del Poeta M (2004). Role of phagocytosis in the virulence of *Cryptococcus neoformans*. *Eukaryotic Cell*.

[193] Monari C, Bistoni F, Vecchiarelli A (2006). Glucuronoxylomannan exhibits potent immunosuppressive properties. *FEMS Yeast Research*.

[194] Nosanchuk JD, Casadevall A (2003). The contribution of melanin to microbial pathogenesis. *Cellular Microbiology*.

[195] Monga DP (1981). Role of macrophages in resistance of mice to experimental cryptococcosis. *Infection and Immunity*.

[196] Osterholzer JJ, Milam JE, Chen GH, Toews GB, Huffnagle GB, Olszewski MA (2009). Role of dendritic cells and alveolar macrophages in regulating early host defense against pulmonary infection with *Cryptococcus neoformans*. *Infection and Immunity*.

[197] Zaragoza O, Taborda CP, Casadevall A (2003). The efficacy of complement-mediated phagocytosis of Cryptococcus neoformans is dependent on the location of C3 in the polysaccharide capsule and involves both direct and indirect C3-mediated interactions. *European Journal of Immunology*.

[198] Levitz SM, DiBenedetto DJ (1989). Paradoxical role of capsule in murine bronchoalveolar macrophage-mediated killing of *Cryptococcus neoformans*. *Journal of Immunology*.

[199] Casadevall A, Pirofski LA (1999). Host-pathogen interactions: redefining the basic concepts of virulence and pathogenicity. *Infection and Immunity*.

[200] Denning DW (1998). Invasive aspergillosis. *Clinical Infectious Diseases*.

[201] Greenberger PA (2002). Allergic bronchopulmonary aspergillosis. *Journal of Allergy and Clinical Immunology*.

[202] Lin SJ, Schranz J, Teutsch SM (2001). Aspergillosis case-fatality rate: systematic review of the literature. *Clinical Infectious Diseases*.

[203] Paoletti M, Rydholm C, Schwier EU (2005). Evidence for sexuality in the opportunistic fungal pathogen Aspergillus fumigatus. *Current Biology*.

[204] Galagan JE, Calvo SE, Cuomo C (2005). Sequencing of Aspergillus nidulans and comparative analysis with *A. fumigatus* and *A. oryzae*. *Nature*.

[205] O’Gorman CM, Fuller HT, Dyer PS (2009). Discovery of a sexual cycle in the opportunistic fungal pathogen *Aspergillus fumigatus*. *Nature*.

[206] Dyer PS, O'Gorman CM (2012). Sexual development and cryptic sexuality in fungi: insights from *Aspergillus* species. *FEMS Microbiology Reviews*.

[207] Sugui JA, Losada L, Wang W (2011). Identification and characterization of an *Aspergillus fumigatus* “supermater” pair. *MBio*.

[208] Jain R, Valiante V, Remme N (2011). The MAP kinase MpkA controls cell wall integrity, oxidative stress response, gliotoxin production and iron adapatation in *Aspergillus fumigatus*. *Molecular Microbiology*.

[209] Cramer RA, Perfect BZ, Pinchai N (2008). Calcineurin target *CrzA* regulates conidial germination, hyphal growth, and pathogenesis of *Aspergillus fumigatus*. *Eukaryotic Cell*.

[210] Nierman WC, Pain A, Anderson MJ (2005). Genomic sequence of the pathogenic and allergenic filamentous fungus *Aspegillus fumigatus*. *Nature*.

[211] Loftus BJ, Fung E, Roncaglia P (2005). The genome of the basidiomycetous yeast and human pathogen *Cryptococcus neoformans*. *Science*.

[212] Moran GP, Coleman DC, Sullivan DJ (2011). Comparative genomics and the evolution of pathogenicity in human pathogenic fungi. *Eukaryotic Cell*.

[213] Abadio AKR, Kioshima ES, Teixeira MM, Martins NF, Maigret B, Felipe MSS (2011). Comparative genomics allowed the identification of drug targets against human fungal pathogens. *BMC Genomics*.

